# Tributyltin chloride (TBT) induces RXRA down-regulation and lipid accumulation in human liver cells

**DOI:** 10.1371/journal.pone.0224405

**Published:** 2019-11-11

**Authors:** Fabio Stossi, Radhika D. Dandekar, Hannah Johnson, Philip Lavere, Charles E. Foulds, Maureen G. Mancini, Michael A. Mancini

**Affiliations:** 1 Department of Molecular and Cellular Biology, Baylor College of Medicine, Houston, TX, United States of America; 2 Integrated Microscopy Core, Baylor College of Medicine, Houston, TX, United States of America; 3 GCC Center for Advanced Microscopy and Image Informatics, Houston, TX, United States of America; 4 Center for Precision Environmental Health, Baylor College of Medicine, Houston, TX, United States of America; 5 Department of Pharmacology and Chemical Biology, Baylor College of Medicine, Houston, TX, United States of America; 6 Dan L. Duncan Comprehensive Cancer Center; Baylor College of Medicine, Houston, TX, United States of America; 7 Center for Translational Cancer Research, Institute of Biosciences and Technology, Texas A&M University, Houston, TX, United States of America; The Ohio State University, UNITED STATES

## Abstract

A subset of environmental chemicals acts as “obesogens” as they increase adipose mass and lipid content in livers of treated rodents. One of the most studied class of obesogens are the tin-containing chemicals that have as a central moiety tributyltin (TBT), which bind and activate two nuclear hormone receptors, Peroxisome Proliferator Activated Receptor Gamma (PPARG) and Retinoid X Receptor Alpha (RXRA), at nanomolar concentrations. Here, we have tested whether TBT chloride at such concentrations may affect the neutral lipid level in two cell line models of human liver. Indeed, using high content image analysis (HCA), TBT significantly increased neutral lipid content in a time- and concentration-dependent manner. Consistent with the observed increased lipid accumulation, RNA fluorescence *in situ* hybridization (RNA FISH) and RT-qPCR experiments revealed that TBT enhanced the steady-state mRNA levels of two key genes for *de novo* lipogenesis, the transcription factor *SREBF1* and its downstream enzymatic target, *FASN*. Importantly, pre-treatment of cells with 2-deoxy-D-glucose reduced TBT-mediated lipid accumulation, thereby suggesting a role for active glycolysis during the process of lipid accumulation. As other RXRA binding ligands can promote RXRA protein turnover via the 26S proteasome, TBT was tested for such an effect in the two liver cell lines. We found that TBT, in a time- and dose-dependent manner, significantly reduced steady-state RXRA levels in a proteasome-dependent manner. While TBT promotes both RXRA protein turnover and lipid accumulation, we found no correlation between these two events at the single cell level, thereby suggesting an additional mechanism may be involved in TBT promotion of lipid accumulation, such as glycolysis.

## Introduction

Endocrine disrupting chemicals (EDCs) are a growing class of compounds with highly varied structures and mechanisms of action that interfere with endocrine and metabolic functions by posing as either agonists or antagonists of physiological hormones [1,2]. A subset of these compounds has been termed “obesogens” as they contribute to metabolic imbalance and obesity, especially when exposure occurs at sensitive developmental windows [3–6]. Obesogenic compounds are comprised of many different classes of environmental pollutants and industrial products, including cigarette smoke, estrogenic compounds, flame retardants and plasticizers; however, improved assays to accurately determine if compounds are indeed potential obesogens continue to be in demand [7].

Here, we focused on the prototypical obesogen tributyltin chloride (TBT), a tin-containing chemical (in the organotin class, together with other tributyltin derivatives, [8,9]) that is used as fungicide and heat stabilizer in compositions of polyvinyl chloride, and, along with its metabolites, has been detected in human blood and liver [10,11]. TBT acts as an agonist for two nuclear hormone receptors (NRs), RXRA and PPARG, and, through changes in gene expression, stimulates adipogenesis by inducing their differentiation from pre-adipocytes and/or from mesenchymal stem cells [12–15]. Early-life exposure of mice and rats has been shown to increase lipid accumulation in adipocytes and liver, which persists in adulthood and in future generations [12,16–18], making it a *bona fide* obesogen and an established reference compound. Here, we analyzed, by imaging and high content analysis (HCA), the effects of TBT in human liver cell lines and determined that even picomolar concentrations of TBT, lower than levels found in human samples (~20 nM), cause a glycolysis-dependent increase in lipid content. We delved into TBT mechanism of action and showed that TBT can quickly activate lipogenic target genes, as determined by single molecule RNA FISH and single cell analysis. Interestingly, TBT also affected the levels of its two main target NRs, PPARG and RXRA, but in opposite directions, with PPARG being increased, while RXRA was decreased in a 26S proteasome-dependent manner. Moreover, at the single cell level, RXRA levels did not correlate with lipid content, similar to our previous results in adipocytes where coregulator proteins did not correlate with NR levels and lipid content [19]. In conclusion, we validated that TBT acts as an obesogen in human liver cells through modulation of lipogenic gene expression and PPARG/RXRA levels.

We further propose that human liver cell lines can be used as an additional tool to describe compounds with obesogenic potential with the clear advantage of having a shorter assay length (48–72 hours) as compared to more traditional 3T3-L1 adipogenesis assay (14 days, [13]).

## Materials and methods

### Cells and reagents

HepaRG and HepG2 cells were obtained from BCM cell culture core that routinely validates cell line identity for customers by genotyping. They were cultured in Williams E media (HepaRG) or DMEM (HepG2) with 10% FBS and L-glutamine for no more than 6 passages. Cells are routinely monitored for mycoplasma contamination by DAPI labeling and always resulted negative. LipidTox and AlexaFluor conjugated secondary antibodies (used at a 1:1000 dilution) are from ThermoFisher, and RXRA and PPARG antibodies are from ActiveMotif (used at 1:1000 dilution). Tributyltin chloride (S1A Fig) and 2-deoxy-D-glucose are from Sigma. MG132 and T0070907 are from Tocris.

### Immunofluorescence and lipid staining

Immunofluorescence experiments were completed as previously described [19,20]. Briefly, cells were fixed in 4% formaldehyde in PBS, quenched with 0.1 M ammonium chloride for 10 min, and permeabilized with 0.5% Triton X-100 for 30 min. Cells were incubated at room temperature in 5% non-fat milk in TBST for 1 hour, and then specific antibodies were added overnight at 4°C prior to 30 min of secondary antibody incubation and DAPI staining. Coverslips were mounted in SlowFade Gold, and multiwell plates were imaged in PBS. For lipid staining, a 1:1000 solution of LipidTox Green was added for 30 minutes at room temperature, after quenching, and no permeabilization was performed. In experiments to detect both lipids and RXRA, Triton X-100 was omitted and substituted with 0.1% saponin/3% BSA in PBS for all the steps after quenching.

### RNA FISH

Cells were fixed in 4% purified formaldehyde (Electron Microscopy Sciences) in ribonuclease (RNase)-free PBS for 15 min at room temperature and then permeabilized with 70% ethanol in RNase-free water at 4°C for a minimum of 1 hour [21]. Cells were washed in 1 ml of wash buffer (2x saline sodium citrate [SSC] plus 10% formamide) followed by overnight hybridization with RNA FISH probes at 37°C (Stellaris® probes; LGC Biosearch Technologies) in Stellaris® RNA FISH hybridization buffer (SMF-HB1-10), followed by one change in wash buffer (30 min at 37°C), and a second wash in buffer containing 1μg/ml DAPI (10 min at 37°C) [21]. Vectashield (Vector Laboratories) was used as the mounting medium. All probes were custom designed, synthesized, and labeled with either Quasar 570® or Quasar 670® dyes by LGC Biosearch Technologies.

### Imaging

Imaging of coverslips was performed in a semi-automated manner on the GE Healthcare DVLive epifluorescence image restoration microscope using either an Olympus PlanApo 60x/1.42 NA or a PlanApo 40x/0.95 NA objective and a 1.9k x 1.9x sCMOS camera. Z stacks (0.25–0.35μm) covering the whole cell (~12μm) were acquired before applying a conservative restorative algorithm for quantitative image deconvolution. Max intensity projections were generated and used for image analysis. High throughput imaging of 96 and 384 well plates (GreinerBioOne) was performed with a Nikon PlanApo 20x/0.75 on a Vala Sciences IC-200 high throughput microscope. All images were saved as 16-bit TIFF greyscale.

### Image analysis and statistics

Image analysis was performed using CellProfiler (www.cellprofiler.org) pipelines (example shown in S1B Fig) for nuclear, cellular and lipid droplets segmentation and measurement extraction. For lipid droplet analysis the following steps were used: images were background subtracted using the rolling ball method; Smooth, IdentifyPrimaryObjects (generates nuclear mask based on smoothed DAPI labeling, its intensity and size), IdentifySecondaryObjects (generates the “cell” mask based on the lipid staining and distance from the nuclear mask–similar to [22]), IdentifyPrimaryObjects (generates the lipid droplet mask by analyzing the Lipidtox stain images using Otsu thresholding and a size filter), IdentifyTertiaryObjects (used to determine the “cytoplasm”, subtracts the nuclear mask from the cell mask), and RelateObjects (relates each lipid droplet mask to the corresponding cytoplasm mask). A much simpler pipeline was used for nuclear protein (*i*.*e*., RXRA and PPARG) measurements as only a nuclear mask was needed. Apoptotic and mitotic cells were filtered out based on DAPI intensity and morphology. One-way non-parametric ANOVA (Kruskal-Wallis) with Dunn’s post-test, non-parametric t-test and curve fitting was performed in GraphPad Prism. Heatmap in Fig 2B was created with Orange Data Mining [23].

### Reverse transcription-quantitative real-time PCR (RT-qPCR)

Briefly, HepaRG cells were treated overnight in six-well dishes with either DMSO vehicle or 50 nM TBT, then total RNA was isolated using RNeasy mini kits (Qiagen). cDNA synthesis and qPCR using Roche Universal Library Probes was performed with comparative Ct data analysis as previously described, using β-actin mRNA as the normalizer [24]. The primers used for *SREBF1* and *FASN* mRNA detection with Roche probes #77 and #11, respectively, are: *SREBF1* Forward, 5’- cgctcctccatcaatgaca-3’; *SREBF1* Reverse, 5’- tgcgcaagacagcagattta-3’; *FASN* Forward, 5’- caggcacacacgatggac-3’; *FASN* Reverse, 5’- cggagtgaatctgggttgat-3’. Student’s t test was used in Excel to define statistical significance of TBT gene inductions as compared to the DMSO control.

## Results

### Tributyltin (TBT) increases neutral lipid content in HepaRG liver cells

**Fig 1:**
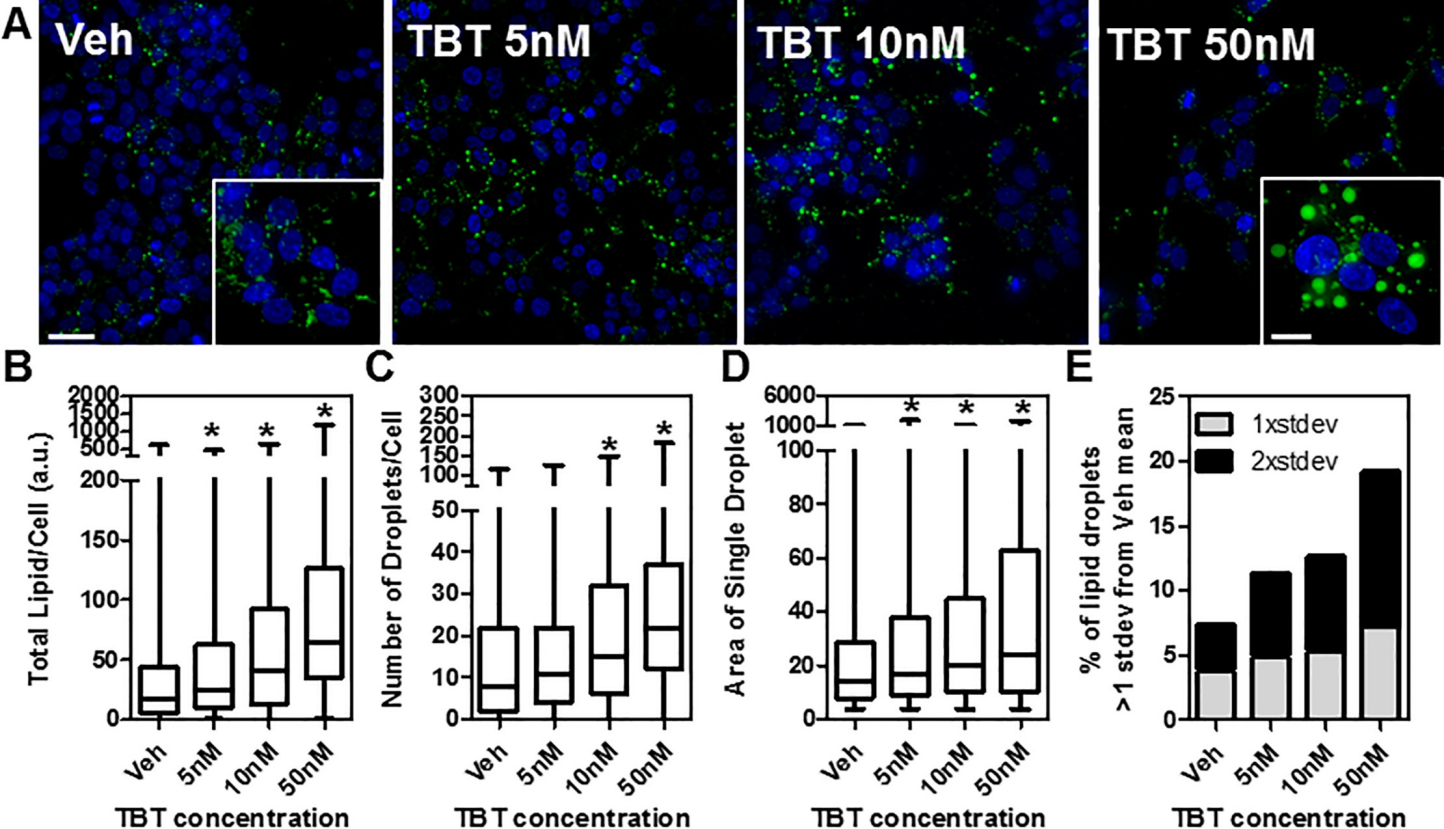
The obesogen tributyltin (TBT) increases lipid content in HepaRG human liver cells. A) HepaRG cells were grown in the presence of TBT (5–50 nM) or vehicle (DMSO) for 14 days, lipid droplets were stained with LipidTox and imaged using deconvolution epifluorescence microscopy; max projection images are shown. B-E) High content image analysis of the effects of TBT at the single cell and single lipid droplet level. n> 400 cells/condition analyzed from two independent experiments. *p<0.05 by ANOVA (Kruskal-Wallis test). Scale bar: 25 μm (10 μm inset).

As TBT was shown to increase lipid content in mice and rat livers after weeks of treatment [12,16–18], we sought to determine if a similar effect was also measurable in human cell line models that could be amenable to high throughput platforms to characterize potential obesogenic chemicals and their mechanism of action in a faster and highly quantitative manner. As a starting point, we treated the human cell line HepaRG, which has been extensively used as a more reproducible model than primary human hepatocytes, while maintaining a gene expression profile comparable to hepatocytes, including xenobiotic enzyme and NRs expression [25]. Mimicking the classical adipogenesis assays in the 3T3-L1 cell model, we treated with TBT chloride for 14 days, and measured neutral lipid content by LipidTox staining, automated imaging and high content image analysis (HCA), as previously described [19,26]. The treatment doses were chosen based on TBT concentration found in human samples [10], and to avoid cytotoxicity. TBT-induced toxicity was evident in HepaRG cells above 100 nM as observed by light microscopy (cell death and pyknotic nuclei). Similarly, TBT-induced cytotoxicity was previously shown to be in the μM range in primary rat hepatocytes [27], and human liver cells [28], plus TBT is a potent biocide affecting mitochondrial potential in several cytotoxicity assays in the ToxCast database (actor.epa.gov/dashboard). We imaged HepaRG cells at both high (60x/1.4NA oil immersion, Fig 1A, inset) and lower (40x/0.95NA air) magnification using an automated image restoration deconvolution microscope (Fig 1A); then, we used image analysis to extract single cell and single lipid droplet data from >400 cells/treatment (S1B Fig).

As shown in Fig 1B–1D, TBT caused a dose-dependent increase of the total lipid content per cell, the number of droplets/cell, and the total area of individual droplets. This conclusion is also evident at the single droplet level when we consider that TBT treatment increased the fraction of droplets that are two standard deviations larger than the mean of vehicle droplets by more than three-fold (Fig 1E).

**Fig 2:**
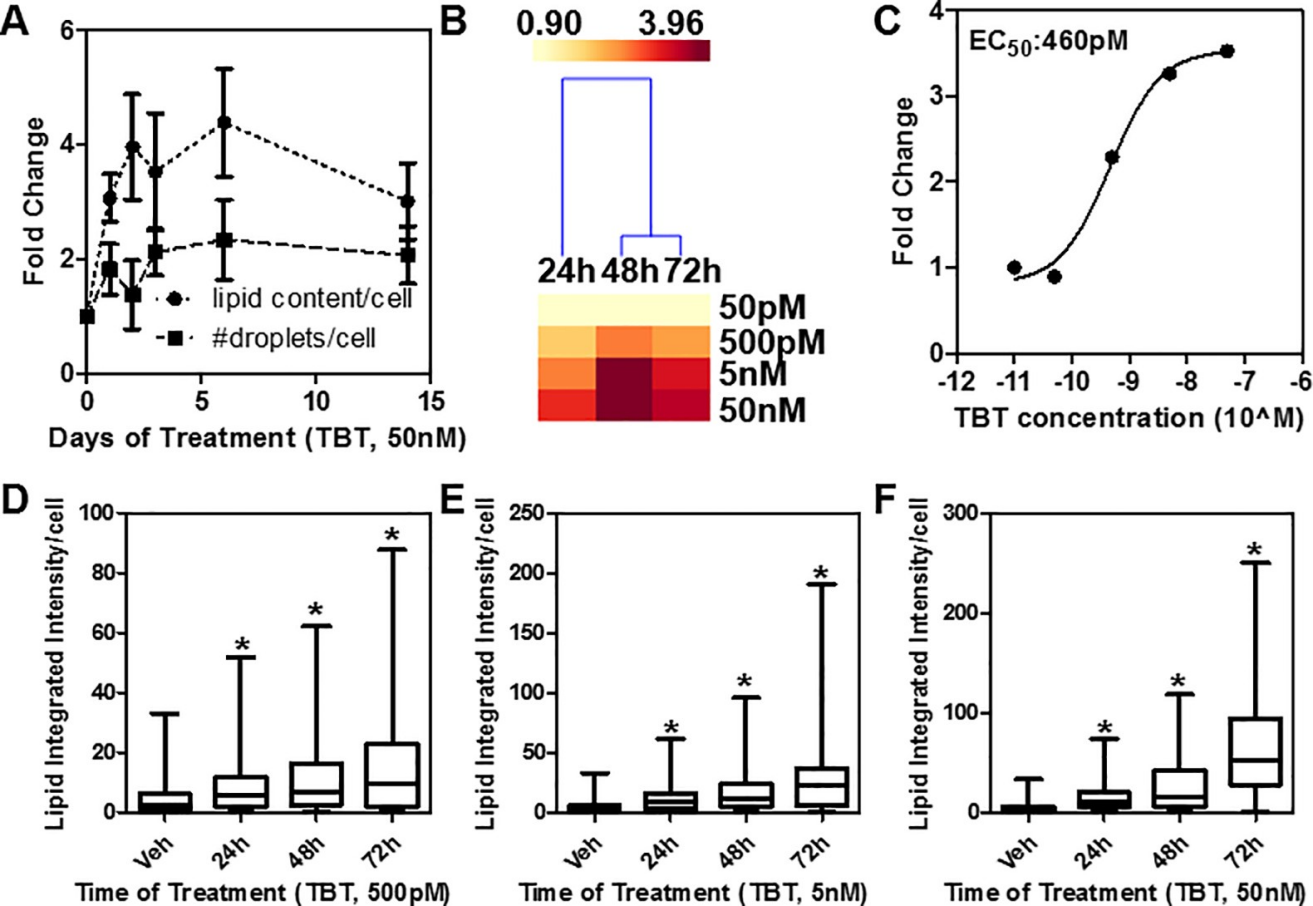
TBT increases lipid content in a time- and dose-dependent manner in HepaRG cells. A) HepaRG cells were grown in the presence of TBT (50 nM) or vehicle for 1 to 14 days and lipid droplets were stained with LipidTox. Y axis is fold change over DMSO treated cells. B) heatmap showing total lipid content/cell after treatment with TBT (50 pM-50 nM) for 1 to 3 days. C) dose-response curve and EC_50_ calculation at the 72 hr time point. D-F) box plots representing single cell data from panel B. *p<0.05 by ANOVA (Kruskal-Wallis test).

We next performed a set of time- and dose-response treatments to determine how fast and at what concentration TBT is capable of increasing lipid content in HepaRG cells (Fig 2).

As shown in Fig 2A, TBT increased significantly both the number of droplets and the total lipid content per cell rapidly (~24 hours), albeit the peak accumulation was recorded at 72 hours. We then performed a four-point TBT dose-response (50 pM to 50 nM) over three days of treatment (Fig 2B). In the heatmap, total lipid content is represented as fold change from vehicle control, with darker red indicating enhancement of lipid levels. More interestingly, as low as 500 pM of TBT (calculated EC_50_: 460 pM, Fig 2C) was sufficient to elicit a measurable and statistically significant response over time, indicating that these cells are highly sensitive to obesogenic insults (Fig 2D–2F). Similar results were also found in HepG2 cells, a hepatocellular carcinoma cell line (S2A and S2B Fig).

**Fig 3:**
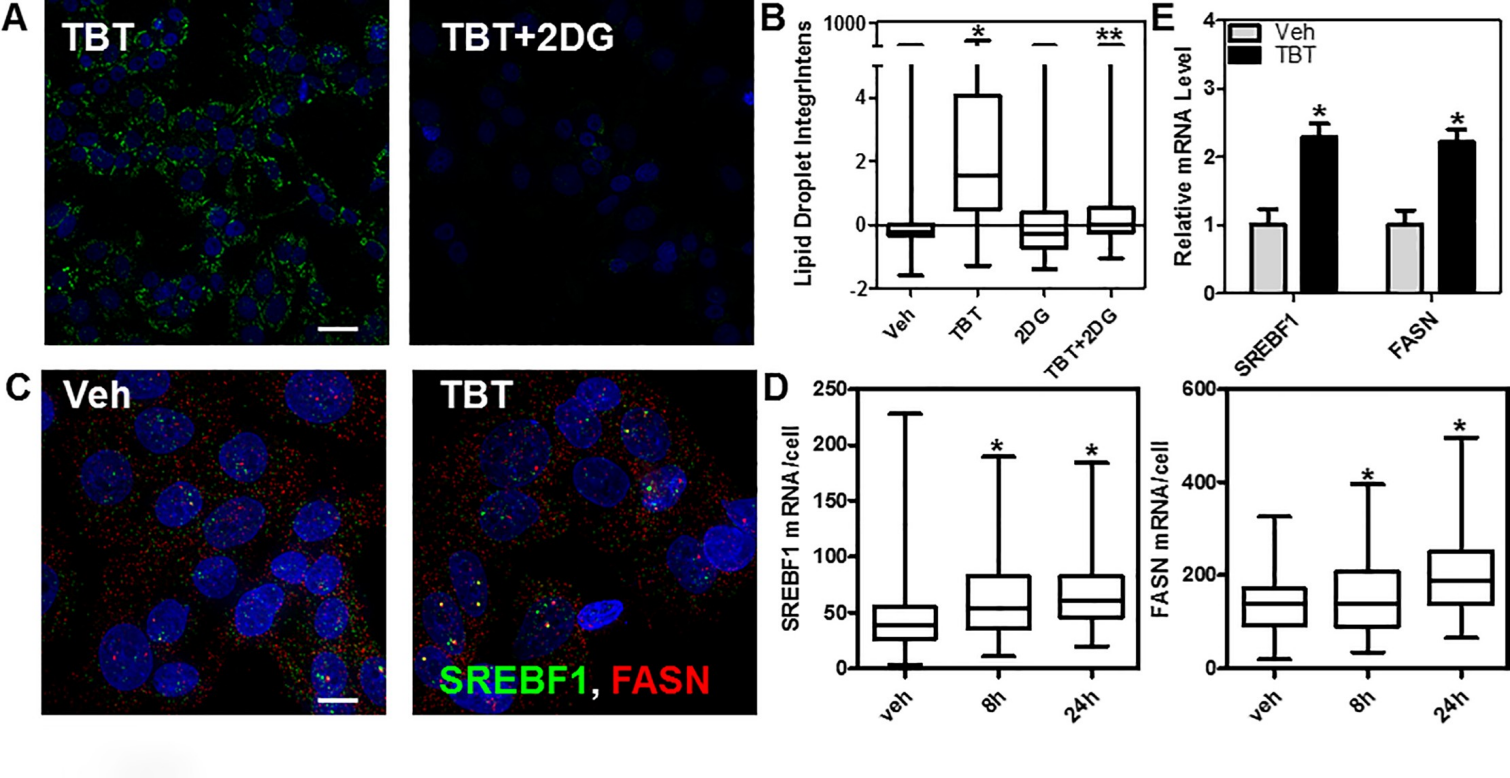
TBT increase in lipid droplets is mediated through glycolysis and activation of some lipogenic genes. A) HepaRG cells were grown in the presence of TBT (50 nM), 2-deoxyglucose (2DG, 2 mM) or vehicle for 3 days, then lipid content was analyzed as in Fig 1. Representative 40x images are shown. Scale bar: 25μm. B) quantification of images from the experiment performed in panel A, data is z-normalized based on vehicle (DMSO) treatment and shown as integrated intensity of lipid droplets/cell. *p<0.01 compared to Vehicle; **p<0.01 compared to TBT. C-D) Single molecule RNA FISH for *SREBF1* and *FASN* was performed in HepaRG cells after 8 and 24 h (shown in the panels) of TBT or vehicle treatment. Images (60x/1.4NA) shown are max projections after deconvolution. In panel D, the number of single RNA molecules/cell was counted and represented as a box plot. Scale bar: 10 μmM. E) RT-qPCR validation of TBT induction of *SREBF1* and *FASN* mRNAs after 24h of 50nM TBT treatment. *p<0.01 compared to Vehicle.

To further explore its mechanism of action, we co-treated cells with TBT and 2-deoxy-D-glucose (2-DG), an inhibitor of glycolysis, which is a central part of the metabolic pathways that lead to *de novo* lipogenesis [29], and measured lipid droplet formation by HCA imaging. As shown in Fig 3A and 3B, 2-DG co-treatment completely abrogated the effect of TBT, clearly showing that the induction of neutral lipid droplet formation is dependent upon active glycolysis.

We also used single molecule RNA FISH (smFISH) [21] to determine the effect of TBT on two putative target genes that are central in *de novo* lipogenesis, *SREBF1* and *FASN*. We treated HepaRG for 8 and 24 hours with 5 nM TBT and measured the number of mature RNAs/cell using automated image analysis. Fig 3C shows representative images of the dual color RNA FISH (*SREBF1* exons in green and *FASN* exons in red) and the quantitation per cell (n>50/condition) highlighting that TBT increases both target genes (Fig 3D). We further confirmed that an overnight exposure of 50 nM TBT increased steady-state *SREBF1* and *FASN* mRNA levels ~2 fold by RT-qPCR (Fig 3E).

### TBT down-regulates RXRA levels in HepaRG cells

**Fig 4:**
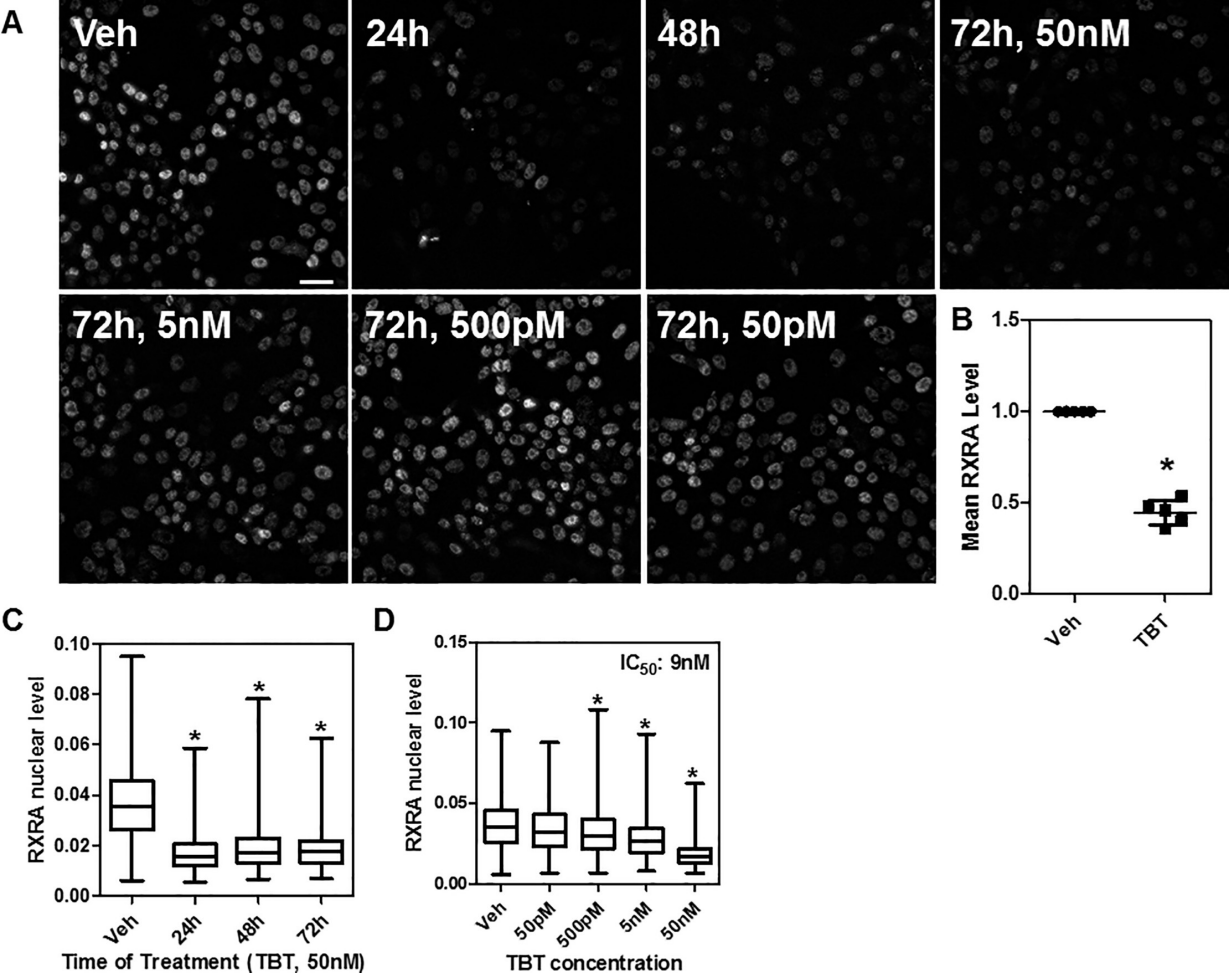
TBT down-regulates RXRA nuclear levels in a time- and dose-dependent manner in HepaRG cells. A) HepaRG cells were grown in the presence of TBT (50 nM) or vehicle for 1 to 3 days, then immunofluorescence was performed using specific RXRA antibody. Representative 20x images (max projection) are shown. Scale bar: 25 μm. B) Fold change of RXRA down-regulation at 72h is reproducible across five independent experiments. C-D) box plots representing single cell data from panel A after high throughput microscopy and image analysis. *p<0.05 by ANOVA (Kruskal-Wallis test with Dunn post test); in panel B a non-parametric t-test was used.

TBT acts as an agonist of two NRs, PPARG and RXRA, to increase adipogenesis [12,13,30,31] with an EC_50_ in the nM range. RXRA is a central transcription factor hub controlling many liver metabolic pathways [32–34] and responds to stimuli (ligand-binding) through heterodimerizing with many different nuclear receptors (*i*.*e*., PPARs, LXRs, FXR, PXR, etc.) involved in liver biology. For these reasons, we quantified RXRA levels by immunofluorescence coupled with high throughput microscopy and single cell image analysis (Fig 4).

**Fig 5:**
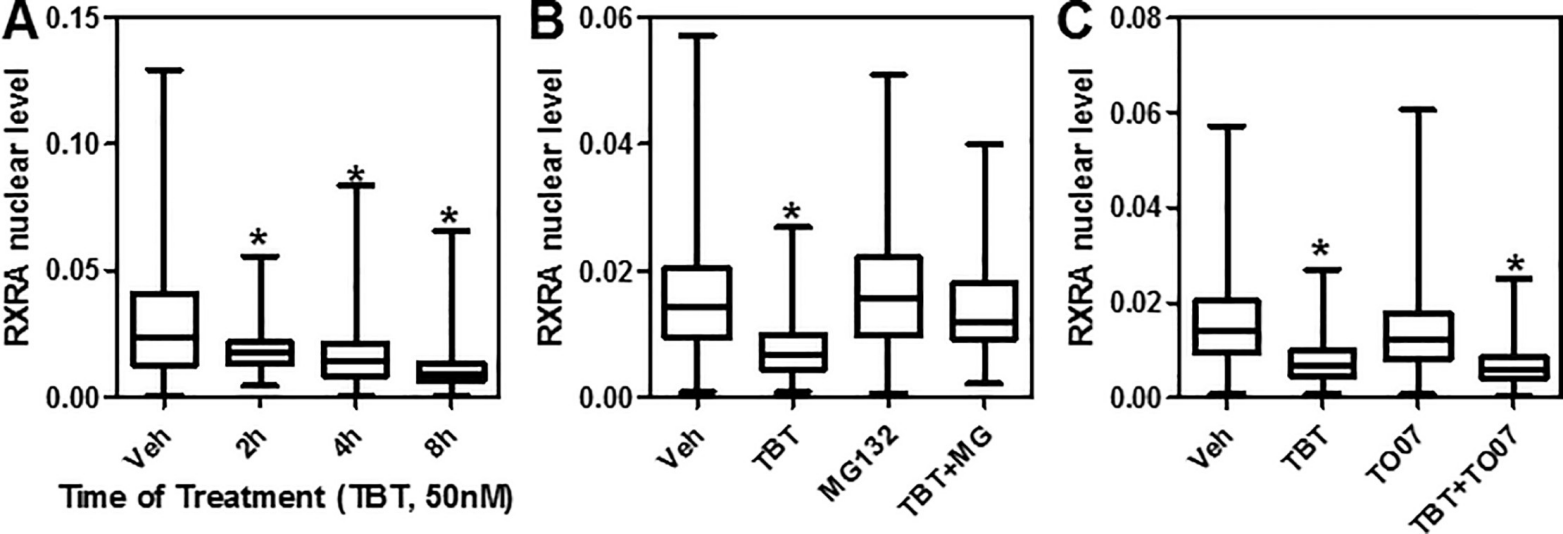
TBT down-regulates RXRA in a fast, proteasome-dependent and PPARG-independent manner. A) HepaRG cells were treated TBT (50 nM) or vehicle for 2–8 hours, then immunofluorescence using specific RXRA antibody was performed and single cell nuclear levels of RXRA measured. B-C) Effect of MG132 and TO070907 on TBT (50 nM, 8 h) down-regulation of RXRA represented as box-plots. *p<0.05 by ANOVA (Kruskal-Wallis test with Dunn post test).

TBT treatment demonstrated a more than two-fold down-regulation of RXRA protein over time and with an IC_50_ of 9 nM, which is compatible with another *in vitro* study of TBT activation of RXRA transcriptional activity [12] (Fig 4B–4D). Similar TBT-mediated RXRA protein down-regulation was observed in HepG2 cells (S2C and S2D Fig). We next tested how rapidly TBT acted on RXRA protein levels and found that only 2 hours of 50 nM TBT treatment were needed to significantly down-regulate RXRA levels (Fig 5A).

Because TBT was shown to covalently bind RXRA [35] and that other synthetic agonist ligands (e.g., LGD1069 and LGD1268) were reported to down-regulate RXRA via the 26S proteasome [36], we checked if TBT-mediated RXRA down-regulation was proteasome dependent. Indeed, the proteasome inhibitor, MG132, blocked the effect of 50 nM TBT at the 8 hour time point (Fig 5B). We also determined that the other TBT binding NR, PPARG, was not involved in the reduction of RXRA as its antagonist T0070907, did not block TBT-mediated RXRA protein turnover (Fig 5C). Interestingly, 50 nM TBT treatment did increase PPARG levels in HepaRG cells (S3 Fig).

### RXRA levels poorly correlate with either lipid content or PPARG at the single cell level

**Fig 6:**
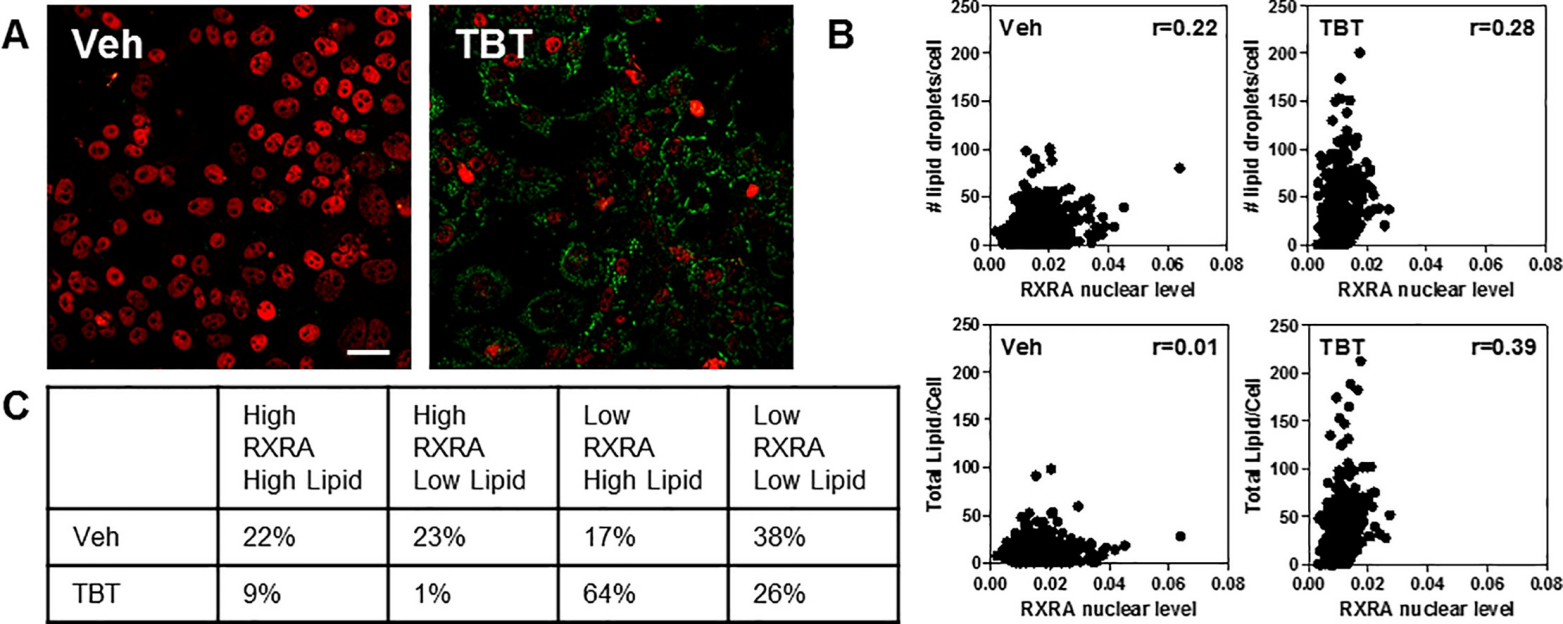
RXRA levels do not correlate with lipid content before or after TBT treatment. A) HepaRG cells were treated TBT (50 nM) or vehicle for 72 h hours, then immunofluorescence using specific RXRA antibody (red), together with LipidTox staining (green), was performed and single cell nuclear levels of RXRA, lipid droplet counts and total lipid content were measured. Scale bar: 25μm. B) Scatter plots showing no significant Spearman r correlation coefficients under both measurement and treatments. C) Table showing the % of cells having either high or low RXRA nuclear level and number of lipid droplets as measured considering the mean values in the vehicle control samples.

Previously, RXRA transcriptional activity mediated by a synthetic agonist correlated with its turnover [36], so we next determined if RXRA levels were linked to lipid content upon TBT treatment of HepaRG cells. We performed a dual labeling experiment for RXRA and lipids after 72 hours of treatment with vehicle or 50 nM TBT. As shown in Fig 6A and 6B, only low correlation (Spearman r <0.4) was observed between RXRA levels and either total lipid content/cell or number of lipid droplets/cell.

Fig 6C further illustrates the opposite effect of TBT on RXRA level and lipid content as shown in the table where we stratified single cell values as high or low (above or below mean values) depending on these two parameters in the vehicle-treated cells. PPARG/RXRA double immunolabeling experiments also showed low correlation (r = 0.4) between the two target NRs in either vehicle or TBT-treated cells, suggesting that levels of the receptors alone may not correlate with activity (S3 Fig), as we have previously shown for NR coregulator proteins in adipogenesis models [19].

## Discussion

The impact of environmental chemicals on metabolic pathways leading to disease (*i*.*e*., obesity and diabetes etc.) has been under extensive investigation for many years [3,6,37]. In particular, a growing class of endocrine disrupting chemicals, termed obesogens [12], has been singled out as a subset of chemicals with potential of increasing adiposity via altering lipid homeostasis and adipogenesis [3,38,39], but also exhibiting additional, systemic effects in other tissues (*i*.*e*., pancreas, liver, muscle, etc.). The effects of obesogens are also dependent upon when exposure occurs, with early-life developmental stages being more susceptible [6,16,40]. Tributyltin, an organotin that has been used as a biocide for many decades, is considered a prototypical tool obesogenic compound. It has a widespread presence in the environment, especially in fresh waters and marine life, due to its use as an anti-fouling reagent in boat paints and other industrial uses [11]. TBT acts through the NRs PPARG and RXRA to increase adipogenesis [12] and, if exposed during pregnancy, the adiposity of offspring is enhanced as manifested in lipid accumulation in liver, testes and adipose tissue [12,40]. Compared to other obesogens, human data on TBT is scarce, although there have been some studies measuring TBT and its metabolites in human blood and liver, likely linked to dietary intake [10,41,42]. The most compelling human epidemiological study comes from assaying placental TBT levels and weight gain in 110 newborn males in Finland, in which a trend towards higher weight gain from birth to 3 months of age with increasing placenta TBT concentration was reported [43].

In this study, we have quantified the effects of TBT on two human liver-derived cell models by both high throughput microscopy and high content analysis. Indeed, TBT was able to induce in a time-, dose- and glycolysis-dependent manner, *de novo* lipogenesis without cytotoxicity, as shown by both lipid droplet analysis and single molecule RNA FISH of two key lipogenic genes. The range of effective concentration (EC_50_ ~500 pM) is compatible with detected levels in human samples while the time course analysis shows significant effects within the first 24–48 hours, making TBT a fast and potent obesogen in human liver cell models. This time/dose effect is highly favorable for screening applications, especially with a growing need to analyze the potential obesogenic effect of compounds and mixtures in a rapid and reproducible manner. We are currently working towards validating high throughput microscopy-based assays for new obesogenic compounds characterization and classification.

As TBT has been shown to interact and modulate both PPARG and RXRA activities *in vitro* and *in vivo* [12,14,30,35], we also wanted to validate this mode of TBT action in the HepaRG model. Interestingly, we found that RXRA is quickly down-regulated at the protein level, via 26S proteasome-dependent pathways, similar to that reported for two other synthetic RXRA agonists [36] while increasing the levels of the PPARG protein, and is potentially relevant to a recent study showing reduction of *RXRA* mRNA in breast cancer cells [44]. Perhaps not surprising [19], we did not find correlations between RXRA and PPARG, or RXRA and lipid content at the single cell level, indicating that other parameters in the cell (*i*.*e*., energy balance, target gene activation, post-translational modifications, etc.) may be more predictive of *de novo* lipogenesis.

In conclusion, we effectively quantified the effects of TBT on *de novo* lipogenesis and RXRA/PPARG levels in two human liver cell models (HepaRG and HepG2) opening the door for the development of fast and sensitive “obesogenic” high throughput imaging-based assays to be used orthogonally with more canonical, long-term experiments (*i*.*e*., 3T3-L1 adipogenesis assay).

## Supporting information

S1 Fig A) structure of Tributyltin chloride (TBT). B) Lipid droplets image analysis pipeline using CellProfiler.
(TIF)

S2 Fig TBT effects on lipid content and RXRA in HepG2 cells. A-B) HepG2 were treated with TBT 50 nM for 72 h and lipid content was measured by LipidTox stain. C-D) HepG2 were treated with TBT 10 nM for 24 h and RXRA immunofluorescence was performed (panel C) and single cell data quantified (panel D). *p<0.05 by ANOVA (Kruskal-Wallis test). Scale bar: 25 μm.
(TIF)

S3 Fig RXRA/PPARG double immunofluorescence analysis in HepaRG cells. A-D) representative 20x images [RXRA (A), PPARG (B), DAPI (C) and merged (D), Scale bar: 50 μm] of HepaRG cells treated with vehicle (left column) or TBT 50 nM (right column) for 72 h. D) quantification of PPARG nuclear intensity at the single cell level represented as a box plot. E-F) RXRA/PPARG correlation analysis (Spearman r) in vehicle vs. TBT treated cells, respectively.
(TIF)

S1 File (XLSX)
