# Supplementary figures and images for: Tributyltin chloride (TBT) induces RXRA down-regulation and lipid accumulation in human liver cells
Source: PLoS One. 2019 Nov 11;14(11):e0224405. doi: 10.1371/journal.pone.0224405 (PMC6844554; doi:10.1371/journal.pone.0224405)

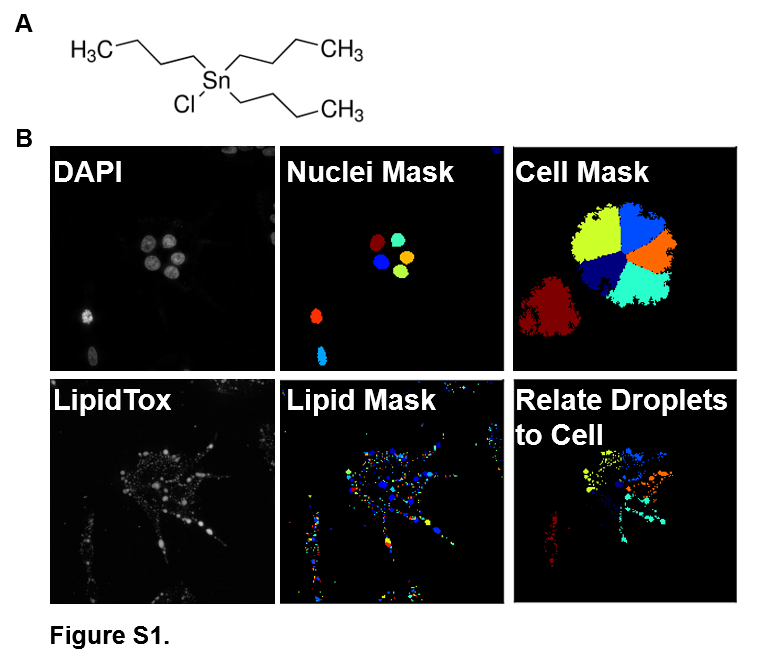

Supplement: S1 Fig — A) structure of Tributyltin chloride (TBT). B) Lipid droplets image analysis pipeline using CellProfiler. (TIF) [file pone.0224405.s001.tif]

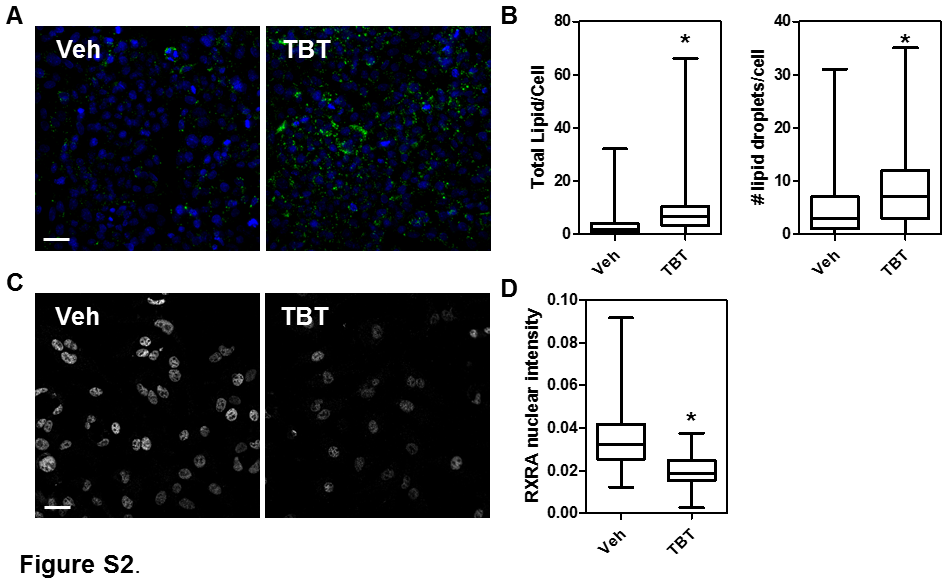

Supplement: S2 Fig — A-B) HepG2 were treated with TBT 50 nM for 72 h and lipid content was measured by LipidTox stain. C-D) HepG2 were treated with TBT 10 nM for 24 h and RXRA immunofluorescence was performed (panel C) and single cell data quantified (panel D). *p<0.05 by ANOVA (Kruskal-Wallis test). Scale bar: 25 μm. (TIF) [file pone.0224405.s002.tif]

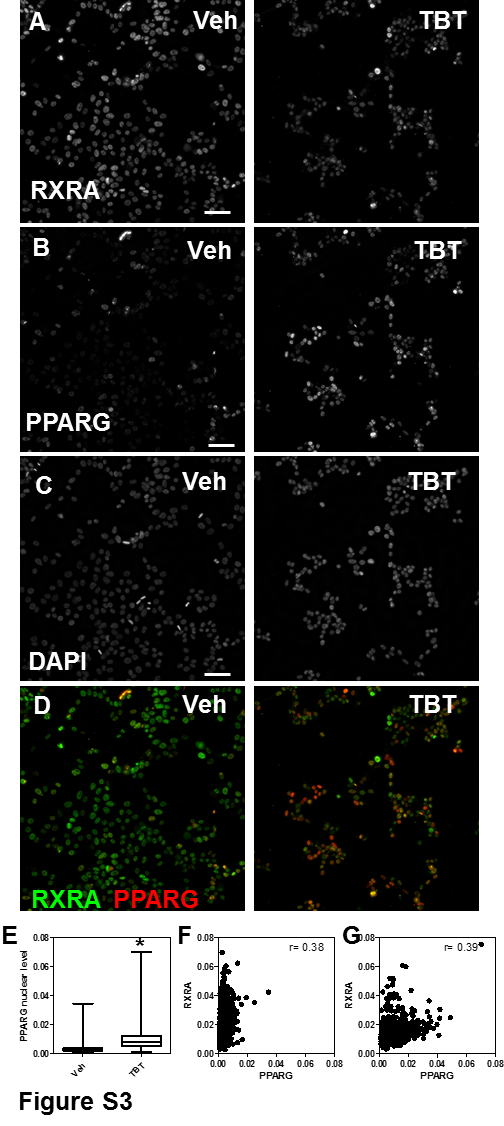

Supplement: S3 Fig — A-D) representative 20x images [RXRA (A), PPARG (B), DAPI (C) and merged (D), Scale bar: 50 μm] of HepaRG cells treated with vehicle (left column) or TBT 50 nM (right column) for 72 h. D) quantification of PPARG nuclear intensity at the single cell level represented as a box plot. E-F) RXRA/PPARG correlation analysis (Spearman r) in vehicle vs. TBT treated cells, respectively. (TIF) [file pone.0224405.s003.tif]
